# Filtered FCS: Species Auto- and Cross-Correlation Functions Highlight Binding and Dynamics in Biomolecules**

**DOI:** 10.1002/cphc.201100897

**Published:** 2012-03-07

**Authors:** Suren Felekyan, Stanislav Kalinin, Hugo Sanabria, Alessandro Valeri, Claus A M Seidel

**Affiliations:** [a] Institut für Physikalische Chemie, Lehrstuhl für Molekulare Physikalische Chemie, Heinrich-Heine-Universität, Universitätsstraße 1Geb. 26.32.02, 40225 Düsseldorf (Germany)

**Keywords:** anisotropy, correlation, energy transfer, fluorescence spectroscopy, spectroscopic methods

## Abstract

An analysis method of lifetime, polarization and spectrally filtered fluorescence correlation spectroscopy, referred to as filtered FCS (fFCS), is introduced. It uses, but is not limited to, multiparameter fluorescence detection to differentiate between molecular species with respect to their fluorescence lifetime, polarization and spectral information. Like the recently introduced fluorescence lifetime correlation spectroscopy (FLCS) [*Chem. Phys. Lett.* **2002**, *353*, 439–445], fFCS is based on pulsed laser excitation. However, it uses the species-specific polarization and spectrally resolved fluorescence decays to generate filters. We determined the most efficient method to generate global filters taking into account the anisotropy information. Thus, fFCS is able to distinguish species, even if they have very close or the same fluorescence lifetime, given differences in other fluorescence parameters. fFCS can be applied as a tool to compute species-specific auto- (SACF) and cross- correlation (SCCF) functions from a mixture of different species for accurate and quantitative analysis of their concentration, diffusion and kinetic properties. The computed correlation curves are also free from artifacts caused by unspecific background signal. We tested this methodology by simulating the extreme case of ligand–receptor binding processes monitored only by differences in fluorescence anisotropy. Furthermore, we apply fFCS to an experimental single-molecule FRET study of an open-to-closed conformational transition of the protein Syntaxin-1. In conclusion, fFCS and the global analysis of the SACFs and SCCF is a key tool to investigate binding processes and conformational dynamics of biomolecules in a nanosecond-to-millisecond time range as well as to unravel the involved molecular states.

## 1. Introduction

Fluorescence correlation spectroscopy (FCS) is frequently used to monitor molecular interactions (binding and unbinding), chemical reaction kinetics and diffusion constants of fluorescent molecules.^1^–^9^ FCS, developed in the early 1970s,^1^, ^10^ improved significantly in the 1990s due to better optics and excitation sources.^11^, ^12^ Regardless of its ultrasensitive detection, complexities arise when taking into account a mixture of fluorescent molecular species with different brightness and different diffusion constants, in particular when the diffusion constants are similar.^13^ Furthermore, scatter background is known to reduce the accuracy of determination of the true number of molecules in the observation volume.^14^ Several ideas to overcome these problems have been implemented in literature. One approach is to combine pulsed excitation and time-resolved fluorescence detection with FCS and time-gate the fluorescence signal in the nanosecond range. This approach can be used to suppress light scattering, but also it can help to separate a dye with a short fluorescence lifetime with respect to a dye with a long fluorescence lifetime.^15^–^21^ However, one major disadvantage of the time-gate approach is that all photons arriving outside the time-gate are discarded in the analysis and becomes too subjective in definition. Another disadvantage is that it cannot fully remove unwanted signal because of “crosstalk” into time gate.

Recently, a different strategy using different fluorescence lifetimes of dyes was proposed and named fluorescence lifetime correlation spectroscopy (FLCS).^22^–^25^ In this case, two independent FCS curves for two different dyes, with different lifetime decay, were extracted from a mixed solution without ignoring any detected photons. The authors describe the method as being “similar to recording a complete spectrum and deconvoluting it into the different spectral contributions from the various emitting species”. One of the advantages of this method is that there is no lost signal. In addition, one can discriminate detector after pulsing from the fluorescence signal.^23^ But one of the most important advantages is that one can obtain FCS curves for different dyes. This method can be generalized for more than two dyes, the only requirement is that they have sufficiently distinct fluorescence lifetimes.^22^ Rüttinger et al.^26^ demonstrated that diffusion times differing only 25 % from each other can be resolved by FLCS. Various applications of FLCS and fluorescence lifetime crosscorrelation spectroscopy (FLCCS) demonstrated the usefulness of this technique for characterizing species-specific diffusion times and biomolecular binding processes in vitro,^27^, ^28^ in live cells^29^ and at a light absorbing interface,^30^ which employed fluorescence lifetime tuning to study diffusion of BODIPY-tail-labeled lipid in distinct lipid bilayer environments. Moreover, FLCS was applied to study the protonation kinetics of fluorescent dyes in aqueous buffers.^31^

The requirement of FLCS, that fluorescence decays are sufficiently distinct due to different fluorescence lifetimes, becomes one of its major limitations. In practice—from our experience—“sufficiently different decay times” means at least 1.0–1.5 ns difference for efficient separation. This corresponds to 20–30 % change in lifetimes of fluorescent dyes, commonly ranging from 1 to 5 ns. Therefore, this requirement significantly restricts dye selection, the number of species, which can be studied in parallel, and the type of problems that could be studied.

The methodology proposed in this work, fFCS, benefits from multiparameter fluorescence detection (MFD).^16^, ^32^, ^33^ Using single-color excitation and MFD, the collected signal is split into several detection channels at different spectral windows considering their parallel and perpendicular polarizations relative to linearly polarized excitation laser beam. In addition to fluorescence lifetime and time resolved anisotropy we can also utilize the spectral differences of species as additional parameter to further improve the distinction between molecular species in a mixture. Two or three dimensional filtering (lifetime and anisotropy, lifetime and color, or lifetime, anisotropy and color) makes this method more precise and selective, for example, 16 species have been studied by Widengren at al.^33^ In particular, it becomes possible to “deconvolute” FCS curves for the contribution of different molecular species even when they have very similar or equal lifetimes. The only requirement is that they differ sufficiently in one parameter or in a combination of parameters if their individual differences are small. Here we demonstrate the case in which a difference of 0.2 ns in rotational correlation times is enough to separate species even when their fluorescence lifetimes are identical.

We tested fFCS for two cases: 1) To characterize the accuracy of fFCS, we simulated the dynamic binding of two biomolecules (e.g. labeled ligand and unlabeled receptor). From fFCS, we extracted what we call the species auto-correlation function (SACF) and species cross-correlation function (SCCF) to quantitatively resolve species fractions in dynamic equilibrium and recover rate constants with relative error less than 2 % in average. The separation of species is possible even in the extreme case where the two biomolecules have the same fluorescence lifetime, but differ by rotational correlation time. Time resolution of sub-microseconds can be achieved. 2) Furthermore, we applied this methodology to recent experimental data^34^ to extract kinetic information about conformational transition between the open and closed state of the protein Syntaxin1 with high temporal resolution. In this case, separation of conformers is maximized by the differences in lifetime and anisotropy using single molecule Förster Resonance Energy Transfer (smFRET) data. Finally, the requirements for an optimal experimental realization of fFCS are discussed.

Combining FRET and FCS has been attempted before;^35^–^37^ however, there are multiple artifacts that complicate the analysis and limit its use. For example, it is almost impossible to avoid donor-only (d-only) labeled species in mixture with donor and acceptor (DA) labeled species. On top of this, one can have photophysical processes, such as triplet formation, *cis*–*trans* isomerization, and donor quenching. Therefore, the detection of small FRET efficiency changes due to conformational transitions in FRET-FCS is a very difficult task due to poor contrast. Nevertheless, with fFCS in principle these complexities can be solved. Additionally, one could apply fFCS in other variations of single molecule FRET experiments, such as pulsed excitation with several lasers referred to as nsALEX^38^ and PIE^39^ (or see article by Kudryavtsev et al. published in this same issue of *ChemPhysChem*^39^), and to assure that the acceptor dye is functional and in this way increase selectivity and confidence that the monitored species are relevant to the study.

Another advantage of fFCS is to accurately separate pure fluorescence from background signal, in particular when measuring pm concentrations in single-molecule experiments. We show in this report, that fFCS is successfully applied to obtain correlation curves with correct amplitudes from single molecule data.

In all, fFCS allows one to extract all possible auto- and cross-correlation functions that could be used to study a wide variety of processes including dynamic binding and conformational dynamics of proteins.

## 2. Results

### 2.1. Finding Optimal Filters

The auto-correlation function allows direct assessment of the diffusion constant *D*. If only diffusion of a single species is considered, the amplitude at zero time of the autocorrelation function *G*(0), allows one to determine the mean number of molecules *N* in the detection volume, *V_det_*, or the concentration, *c*, if the parameters of detection volume are known [Eq. (1)]:


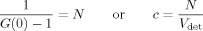
(1)

Unfortunately, the situation becomes significantly more complicated if more than one molecular species are simultaneously present in solution. Considering a mixture of *n* species, with corresponding brightnesses 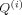, diffusion constants *D*^(*i*)^ and fractions *x*^(*i*)^, *i=1*,…, *n*, it is most convenient to define the autocorrelation function as^40^ [Eq. (2)]:


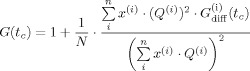
(2)

where *c*^(*i*)^ concentration, 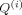 brightness and 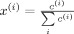 fraction of species *i*, and *N* corresponds now to the total number of molecules. The normalized diffusion term of species *i* [i.e. 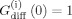] is given by Equation (3):


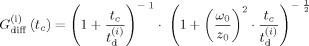
(3)

This model assumes a three-dimensional (3D) Gaussian-shaped volume element with spatial distribution of the detection probabilities 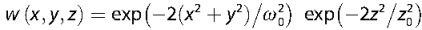. The 1/*e*^2^ radii in *x*, *y* and in *z* directions are denoted by *ω*_0_ and *z*_0_, respectively. For one-photon excitation, the characteristic diffusion time 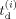 can be used to estimate the diffusion coefficient 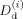 by 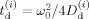.

A simple relation like Equation (1) cannot be found for multiple species. Even if one knows the brightness 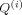 for each species, the diffusion coefficients 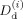
still have to be significantly distinct for successful extraction of *c*^(*i*)^ values, highlighting the need of a proper methodology that could differentiate species in a mixture. In ref. 22 it was proposed to use the differences in fluorescence lifetime to separate molecular species by time-correlated single-photon counting (TCSPC).^41^, ^42^ For simplicity, we will describe how to generate optimal filters for separating two molecular species with mono color detection split for polarization. However, fFCS can be easily extended to account for spectral differences. In addition, fFCS can in principle be used to separate more than two species.

To fully use the anisotropy information a relationship between the total intensities in the parallel and perpendicular detection channels is kept. Therefore the methodology to find the proper filters by minimizing the relative errors proposed in ref. 22 needs to be optimized. Having this in mind, there are in principle three possible scenarios to generate filters: Scenario 1: single detector, single filter (^*1d–s*^*f*). Scenario 2: two detectors, independent filters (^*2d–in*^*f*). Scenario 3: two detectors, global filter (^*2d–gl*^*f*). We found that scenario 3 is most efficient in separating the species. It is described in more detail below. The theory behind scenario 1 was described in ref. 22, and in Section 4.1 we present the case for scenario 2.

In the case of MFD, where the situation is not as trivial as in the case of one-channel detection, the fluorescence signal from the mixture is divided into its parallel and perpendicular components and into two spectral ranges. Each registered photon emitted by *i*th species is detected with probabilities 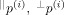 in either parallel or perpendicular detection channel, and certain relations between 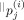 and 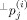 exist due to specific anisotropy decays. If these relationships are disregarded (as done in scenarios 1 and 2), the anisotropy information is lost. To solve this problem, in scenario 3 we stack the TCSPC histograms of the perpendicular over the parallel detection channels and apply a global normalization to the stacked TCSPC histogram. As result, the corresponding probabilities of each *i*th species are obtained as [Eq. (4)]:


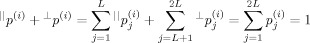
(4)

where the parallel channel probability is [Eq. (5)]:


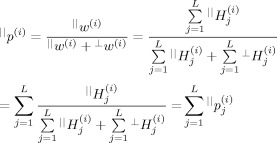
(5)

and similarly, the perpendicular channel probability is [Eq. (6)]:


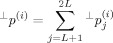
(6)

Thus, 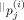 and 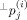 represent the conditional probabilities to register a photon in the *j*th bin of parallel or perpendicular TCSPC channels, provided that photon is emitted by *i*th species. To differentiate from what is done in Section 4.1, here we define 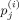 as the stacked conditional probability of length 2*L*. This is done to maintain the anisotropy information. To account for spectral differences (color detection) the histograms are stacked accordingly.

The total number of registered photons 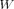 is the sum of photon numbers emitted by all species in mixture solution [Eq. (7)]:


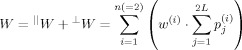
(7)

where 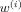 is the photon counts of species *i*. Instead of two measured decay histograms (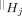 and 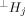) of the two species in the mixture we consider one bimodal decay histogram 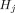 with total number of the stacked TCSPC channels 2*L* and the conditional probability distribution 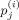 for the *i*th species. This bimodal decay distribution can be defined as [Eq. (8)]:


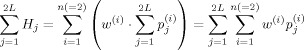
(8)

Where [Eq. (9)]:


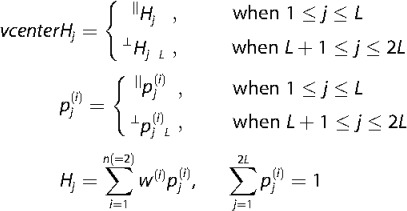
(9)

Using the global definition in Equation (8), two bimodal filters 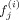, *i*=1, 2, will be generated with the property [Eq. (10)]:


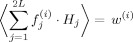
(10)

where the brackets denote averaging over long time of measurements. Filters 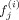 are obtained by minimizing relative errors^43^ for parallel and perpendicular detection channels simultaneously [Eq. (11)]:


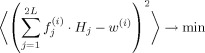
(11)

The idea of one bimodal decay histogram 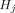 and two conditional probability distributions 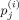, one for each species with total number of stacked TCSPC channels 2*L*, makes it possible to calculate what we refer to as the species auto- (SACF) and cross-correlation (SCCF) function in a similar fashion to ref. 22. However, we need to transform our raw data streams into a modified format where the TCSPC channels of both detectors are stacked in a single array with length 2*L*. In this way, the SCCF 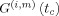 between species *i* and *m* is [Eq. (12)]:


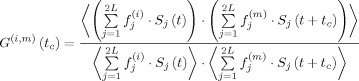
(12)

where 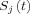 is the signal in the *j*th stacked TCSPC channel array of the total signal at measurement time *t* and 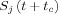 is the signal in the stacked TCSPC channel *j*th at measurement time 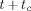. The SACF is defined for the case of *i=m* while SCCF corresponds to different species *i*≠*m*. In both cases, the orthonormality relationship [Eq. (13)]:


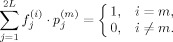
(13)

is satisfied. Then, the species auto- and cross-correlation functions 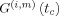 averaged over an infinite number of measurements or over sufficiently long measurement time (≫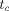) are [Eq. (14)]:


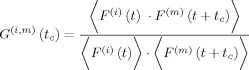
(14)

where 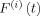 and 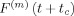 are pure fluorescence signals from the molecular species of *i*th and *m*th type. In a two-state system, 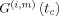 and 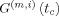 are equal. Thus, since the anisotropy differences are counted in addition to lifetimes, it becomes possible to highlight any dynamic process between two species in mixture solution if they differ in rotational correlation time or/and lifetime. In contrast to standard correlation curves the amplitude of dynamic term per molecule in species cross correlation function is equal to −1, like in an antibunching term that will be discussed in Section 2.2.1. One can understand the shape of SCCF as a probability distribution of interconversion between species: for very short *t*_c_ this probability is nearly equal to zero (SCCF starts at baseline level) and gets higher for larger *t*_c_ proportional to relaxation time of dynamics. Finally, everything is limited by diffusion time and probability to observe a transition is dropping to baseline level. If dynamics between species is missing then SCCF is showing no additional correlation amplitude above baseline.

In comparison to FCS, the species auto-correlation function (SACF) as defined in Equation (12) with *i*=*m* assumes that other species are essentially non-fluorescent. In particular, it can be considered a background/scatter free autocorrelation function if a scatter filter was included. Then, the amplitude of the SACF is inversely related to the true number of molecules with the specific lifetime and polarization characteristics given by the filters. And in the same way as in standard FCS, at small lag times species are highly correlated and for longer correlation times everything is limited by diffusion showing a decrease of amplitude.

Let us consider an extreme case where two molecular species are simulated for typical conditions in single-molecule experiment: both species have the same fluorescence lifetime (*τ*_G_^(1)^=*τ*_G_^(2)^=4.0 ns), same brightness *Q*^(1)^=*Q*^(2)^=150 kHz, but differ only by rotational correlation times (*ρ*^(1)^=0.1 ns, *ρ*^(2)^=0.3 ns). Also, both species have the same diffusion time (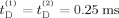). The average number of simulated molecules in the observation volume was 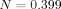 (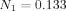 and 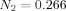). Details on the Brownian dynamics simulator can be found in Section 4.2. The chosen brightnesses are typical for FCS using confocal microscopy.^44^–^46^ By applying additives like triplet or radical quenchers the signal can be further increased.^47^

**Figure 1:**
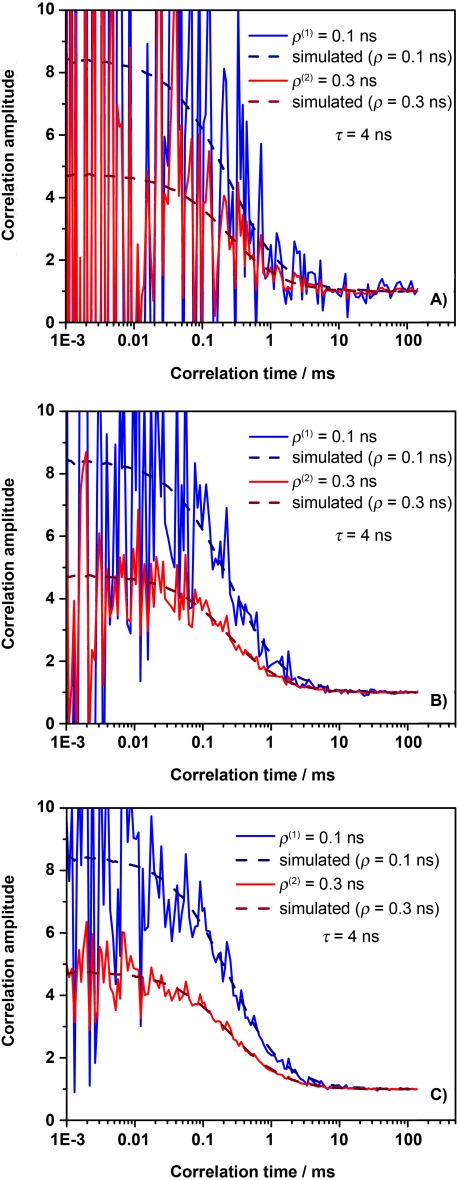
Comparison of SACF obtained by using three possible filter-generation scenarios. The species in the simulated mixture have the same lifetime but different rotational correlation times. Both species have the same brightness *Q*^(1)^=*Q*^(2)^=150 kHz, and identical diffusion times *t*_d_^(1)^=*t*_d_^(2)^=0.25 ms. The number of molecules per species are *N*^(1)^=0.133, *N*^(2)^=0.266, respectively. Dark counts=0.2 kHz, and scatter (IRF)=2.5 kHz rates were considered. The lifetime of both species is *τ_G_*^(1)^=*τ_G_*^(2)^=4.0 ns, and the respective rotational correlation times are *ρ*^(1)^=0.1 ns, *ρ*^(2)^=0.3 ns. The total simulated experiment duration is 2479 s. The SACF for each species are shown in solid blue and red lines for species 1 and 2 respectively. For comparison the simulated correlation function of each species are shown as dashed lines in dark blue and wine for species 1 and 2. A) The calculated SACF by Equation (12) with no split in polarization (scenario 1: assuming 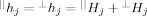 as a case imitating single detection channel experiment). The same decay pattern is used for both detection channels and correspondingly the same filters are applied for parallel and perpendicular detection channels to mimic the one detector case. The correlations show poor statistics with some degree of separation between species. B) scenario 2: SACF from Equation (12) using two detectors, independent filter generation for each detection channel and stacking them afterwards. The separation is better compared to the single detector case. C) SACF of scenario 3 using Equation (12) for two detectors and global filter generation. The separation of species is obvious to the eye, and the estimated error in the recovered parameters is within 2 %. A detail error analysis of recovered parameters is done in Section 2.2.

This plausible experimental situation is simulated to emphasize that only differences in anisotropy can be used to separate species. The same simulation data set is used to determine the SACFs of the three different scenarios of filter generation. In all cases, exactly the same number of photons is correlated. The detection is always considered in two detection channels (parallel and perpendicular). Figure 1 shows the comparison of calculated SACF by Equation (12) curves for the three difference scenarios. In all panels solid blue and red lines correspond to the SACF for species 1 and 2 respectively. On top the modeled correlation curves are shown as dashed lines.

Scenario 1: Single detector, single filter (^*1d–s*^*f*). In this case, Figure 1 A, there is no split by polarization, although polarized excitation was used. To mimic a single detector from the same simulation, we assumed that the total signal is the sum of the two simulated channels 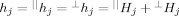. The same 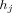 decay pattern is used for both detection channels (parallel and perpendicular) and correspondingly the same filters are applied for parallel and perpendicular detection channels. The conditional probabilities 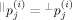 are obtained via Equations (4–6) using 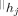 and 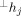. Filters are generated by simultaneously minimizing relative errors in both detection channels [Eq. (11)].

Scenario 2: Two detectors, independent filters (^*2d–in*^*f*). Figure 1 B shows the SACFs using independent filter generation for each detection channel and stacking them afterwards. The conditional probabilities 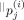 and 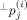
are obtained via Equation (27). 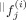 and 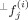 filters are generated by simultaneously and independently minimizing relative errors in both detection channels [Eq. (29)].

Scenario 3: Two detectors, global filter (^*2d–gl*^*f*). When using global filter generation the conditional probabilities 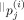 and 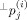
are obtained via Equations (4–6). 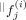 and 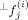 filters are generated by simultaneously minimizing relative errors in both detection channels [Eq. (11)].

The case imitating one detection channel (Figure 1 A) shows poor statistics with some degree of separation between species because of polarized excitation. The separation gets better compared to the single detector imitating case for SACF from two detectors using independent filter generation (Figure 1 B). Compared to Figures 1 A, B, the contrast in Figure 1 C is greater and a clear separation of species is possible, if the anisotropy is properly considered. This is only possible when polarized detection, splitting the fluorescence signal into parallel and perpendicular detection channels, is used like in single molecule MFD setups. In all following results scenario 3 of generating filters is used.

It is worth mentioning that in all cases, the background photons were considered as a third species. The normalized TCSPC histograms (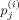) for each species and their corresponding filters according to the three different scenarios are shown in Figure 9. The use of the background filter is an additional standard methodology used for correctly recovering the amplitude of the correlation and was used in all simulated and experimental data analyses.

### 2.2. Results for Polarization-Resolved Simulations

#### 2.2.1. Static/Dynamic Binding Equilibria: Species Auto- and Cross-Correlation Function Separate Fractions

To test the implementation of fFCS, we generated different simulated experiments using a Brownian dynamics approach (see Section 4.2). All fluorescence spectroscopic parameters of molecules freely diffusing through a confocal observation volume were generated. First, we simulated a mixture of molecular species which differ only by their rotational correlation times. This example could represent “static” mixture of free ligand molecules and static complexes (e.g. free labeled DNA and labeled DNA bound to an unlabeled protein as in the case of a binding experiment where the diffusion coefficient does not change significantly). Second, we simulated a mixture of free ligands and complexes, but now we consider a dynamic interconversion between these two species, which may represent a kinetic exchange between a bound and free DNA. In the following we discuss the static case where the exchange relaxation time is much larger than the characteristic diffusion times (*t*_R_≫*t*_d_) and the dynamic case with *t*_R_ smaller or comparable to *t*_d_. A possible application of this example is to study conformational dynamics of biomolecules using a single dye, given that there is a significantly large change in its rotational correlation time.

For the cases of static and dynamic binding equilibria Equation (15), we have simulated mixtures of two species, free ligand, *L*_f_, and the complex, (*LR*), that is, ligand bound by the receptor *R* [Eq. (15)]:


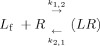
(15)

The relative concentration of receptor to ligand is considered high that the process can be modeled as a pseudo-first-order binding process with the rate constant of association *k*_1,2_*=k_a_*⋅[*R*] and rate constant of dissociation *k*_2,1_. The species fractions *X* in the dynamic equilibrium are defined by Equation (16):


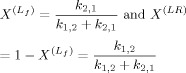
(16)

The inverse of the sum of the rate constants corresponds to the characteristic relaxation time of binding equilibrium *t*_R_ observable in FCS [Eq. (17)]:


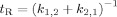
(17)

**Figure 2:**
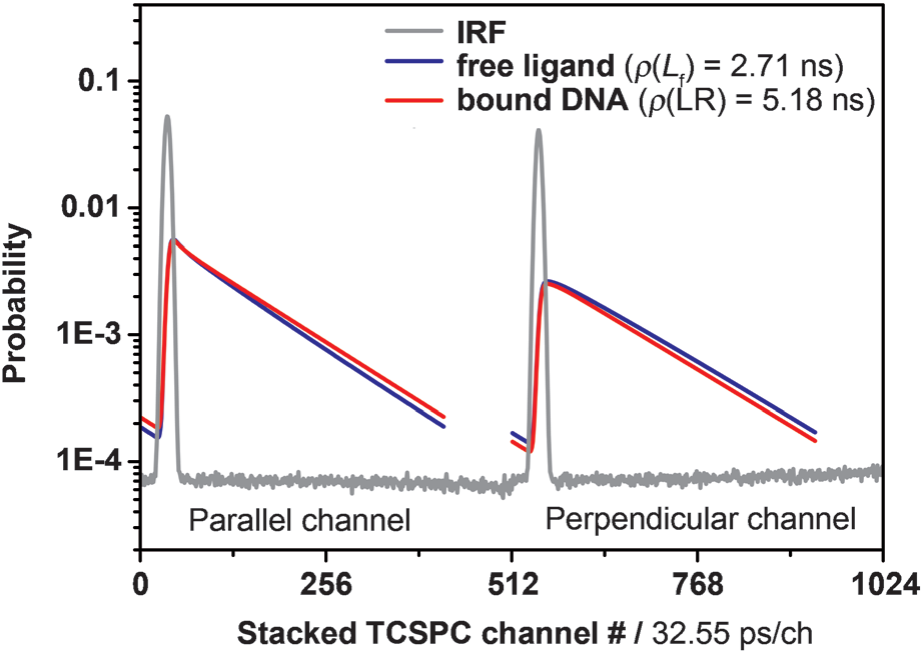
Conditional probabilities of polarization resolved fluorescence decays used in simulations for static and dynamic binding equilibria of two species in buffer: free ligand (*ρ*^(free ligand)^=2.71 ns) and complex (*ρ*^(complex)^=5.18 ns).

For the static case *k*_1,2_=*k*_2,1_=0. In other words, there is no exchange between species. We have used the same 4 ns lifetime for both species but different characteristic rotational correlation times, *ρ*^(free-ligand)^=2.71 ns and *ρ*^(complex)^=5.18 ns, are similar to experimental values from real DNA-protein binding experiments. Both species had the same diffusion time, 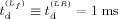, and the same brightness, 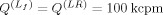, (in order to make the situation very extreme). The concentrations with an average number *N* of molecules in the focus were chosen in the simulations such that they were optimal for the two measurement types: *N*=0.1 for correlation analyses and *N*=0.02 for single-molecule experiments. The generated data files where then filtered with the time and polarization decays shown in Figure 2.

**Figure 3:**
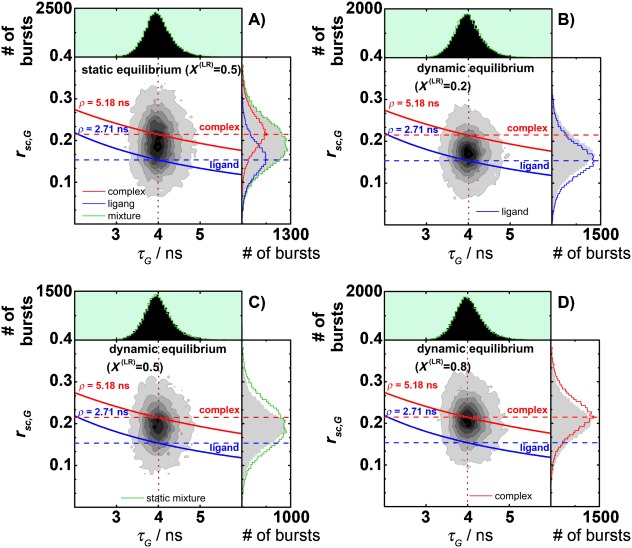
Burstwise analysis of single-molecule events in simulations of an SMD experiment. 2D histograms of fluorescence lifetime (*τ_G_*) distributions on the *x* axis and scatter-corrected anisotropy *r*_sc,G_ on the *y* axis for mixtures of two species [free-labeled ligand (*ρ*^(free ligand)^=2.71 ns) and a complex (*ρ*^(complex)^=5.18 ns)] in buffer. The overlaid red and blue curves show the Perrin equation 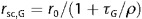 with a fundamental anisotropy *r*_0_=0.375 and the species-specific rotational correlation times *ρ*. A) 50 by 50 percent (*X*^(LR)^=0.5) mixture of static species. B) 80 by 20 percent (*X*^(LR)^=0.2) mixture of dynamic species (*k*_1,2_=2000 s^−1^; *k*_2,1_=8000 s^−1^ or *t*_R_=0.1 ms), C) 50 by 50 percent (*X*^(LR)^=0.5) mixture of dynamic species (*k*_1,2_=*k*_2,1_=2000 s^−1^ or *t_R_*=0.25 ms). D) 20 by 80 percent (*X*^*(LR)*^=0.8) mixture of dynamic species (*k*_1,2_=8000 s^−1^; *k*_2,1_=2000 s^−1^ or *t*_R_=0.1 ms). The 1D lifetime *τ_G_* distributions are overlaid by corresponding ones from 50 by 50 percent static mixture (green line). The 1D *r*_sc,G_ distributions (gray) are overlaid by distributions from 100 % free ligand (blue line), 50 by 50 percent mixture of static species (green line) and 100 % complex (red line).

For direct visual control it is useful to check the behavior of simulated model systems by MFD at single molecule level applying the standard analysis techniques described in refs. 32, 48, 49. In Figure 3 the results of a burstwise analysis are displayed in two-dimensional frequency histograms of scatter corrected anisotropy, *r*_sc,*G*_,^48^ against fluorescence lifetime, *τ_G_*. The corresponding one-dimensional parameter histograms are given as projections. As one can easily see from Figure 3, the lifetime distributions are not affected by dynamics. However, the differences in anisotropy histograms clearly show the expected mixing of states within single burst. As references the 1D *r*_sc,*G*_ distributions (gray bars) are overlaid by distributions obtained from reference conditions: 1) pure free ligand (blue line); 2) pure complex (red line); and 3) mixture with static equilibrium and *X*^(*LR*)^=0.5 (green line). For a static equilibrium (Figure 3 A), the anisotropy of the mixture is nicely described by the sum of the individual components. Considering dynamic binding equilibria with an increasing amount of complex (Figure 3 B–D), the maximum of experimental distribution (gray bars) continuously moves from a lower *r*_sc,*G*_ value (free DNA) to a higher one (100 % complex), which indicates the increasing steady-state fraction of the complex. In Figure 3 C, we see the presence of dynamics by the fact that the gray anisotropy distribution is narrower than the corresponding static equilibrium (green line). However, these observations do not allow us to answer the question of how fast the binding dynamics is. In other words, we need to get a reliable technique to distinguish the dynamic binding from static equilibrium. Something that highlights the interconversion rate is needed, and fFCS provides such information.

**Figure 4:**
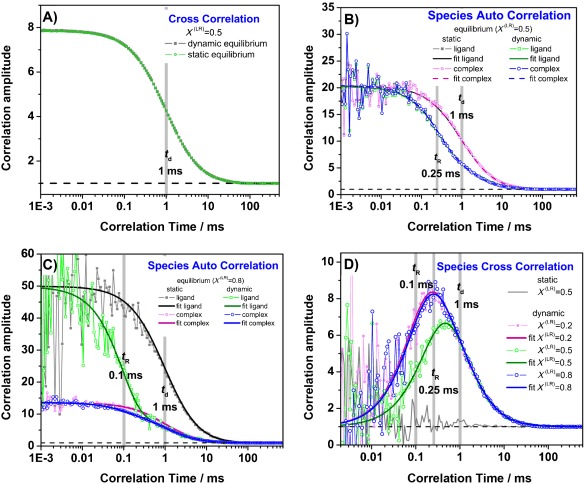
Simulation of an FCS experiment for distinct mixtures of species [free ligand (*ρ*^(free Ligand)^=2.71 ns) and complex (*ρ*^(complex)^=5.18 ns)] in buffer with *N*=0.1. The data generated in the simulation correspond to a measurement time in a real experiment of approximately 12 500 s. The geometric shape of the excitation focus was defined as 3D Gaussian with 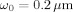 and 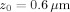. The background signal consists of dark counts=0.2 kHz and scatter=1.8 kHz. The filtered FCS curves were computed by using the filters generated from polarization-resolved decays shown in Figure 2. Comparison of FCCS, SACFs and fit curves for the following cases: A) Overlay of FCCS curves calculated from raw simulated data for 50/50 % static (gray) and 50/50 % dynamic mixtures (green). B) Overlay of SACFs for 50/50 % mixtures: static and dynamic equilibrium, respectively, with (*k*_1,2_=*k*_2,1_=2000 s^−1^ or *t*_R_=0.25 ms). C) Overlay of SACFs for 20/80 % mixtures: static and dynamic equilibrium, respectively, with (*k*_1,2_=8000 s^−1^, *k*_2,1_=2000 s^−1^ or *t*_R_=0.1 ms). D) Overlay of SCCFs for two cases: i) 50/50 % mixtures: static (gray curve) and dynamic (green curve, *k*_1,2_=*k*_2,1_=2000 s^−1^ or *t*_R_=0.25 ms) equilibrium (fit results to *t_R_*=0.246 ms); ii) 80/20 % mixture in a dynamic equilibrium (pink curve, *k*_1,2_=2000 s^−1^, *k*_2,1_=8000 s^−1^ or *t_R_*=0.1 ms). The fit gave *t*_R_=0.096 ms; iii) 20/80 % mixture in a dynamic equilibrium (blue curve, *k*_1,2_=8000 s^−1^, *k*_2,1_=2000 s^−1^ or *t*_R_=0.1 ms). The fit results in *t*_R_=0.099 ms.

The standard FCCS curves (signal in parallel channel versus one in perpendicular) calculated from raw simulated TCSPC data for 50/50 % static and 50/50 % dynamic equilibria are overlaid in Figure 4 A. Two features become immediately apparent—correlation amplitudes are lower than expected and there is no bunching term sensing the dynamic exchange. In the absence of background *G*(0)=10 should be obtained since we know the exact number of particles 0.1 in excitation volume for simulated data. However, the contribution of uncorrelated background from buffer results in lower amplitude of curves (*G*(0)≅7) and wrong *N*_FCS_=0.143. Moreover, regular FCS cannot detect the dynamics in this type of samples, and the decay is sufficiently described by a diffusion term (single gray bar at 1 ms in Figure 4 A). Thus, the absence of any difference may be easily misinterpreted as absolute “identity” of the two studied samples. This is a typical example where classical FCS alone very often overlooks properties of samples, which have been identified as heterogeneous by MFD (Figure 3). In Figure 4 B, we demonstrate that fFCS is free of these uncertainties. Applying the corresponding filter sets for three components in Figure 2 [background (IRF), species 1 (free ligand) and species 2 (complex)] obtained by global error minimization [Eq. (11)], the SACFs were computed using Equation (12). In Figures 4 B,C, the SACFs for static and dynamic equilibria are compared for complex fractions of 50 and 80 percent. The differences are so huge that even without fitting the curves we can note the main important outcomes: 1) Considering a static equilibrium, the SACFs (gray and light magenta curves) have only diffusion terms whereas the SACFs for a dynamic equilibrium (green and blue) decay much faster due to an additional bunching term; 2) the average number of molecules per species is equal in both cases (static and dynamic equilibrium) but all molecules in the mixture are contributing to the diffusion term of the dynamic equilibrium. This is nicely demonstrated for the case of 50 % percent complex (Figure 4 B) by overlaying all SACFs, where the data of the complex (light magenta squares) hide those of the ligand (gray squares). Each of them contains the same particle number in the diffusion term as defined in Equation (18) [1/(*N*(1−*X*^(*i*)^))=20]. We fitted the SACFs with a model function similar in structure to one usually used for fitting the diffusion with a single bunching term [Eq. (18)]:


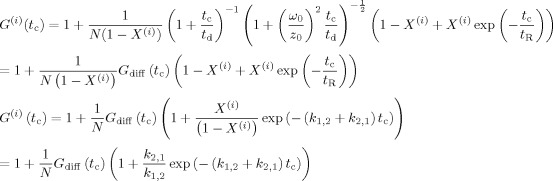
(18)

where *N* is the total number of labeled particles in the excitation volume, *X*^(*i*)^ is the fraction of species *I*, and *t*_R_ is the relaxation time of binding equilibrium (the other parameters are as defined before).

In this model there are no contributions defined by changes in brightness like the singlet–triplet transition case where the molecule is either bright or dark. In this case, the molecule corresponds to species 1 or 2. The fits show the feasibility of this function. The obtained amplitudes agree nicely (error<3 %) with simulations, where the total number of molecules in the focus contributing to diffusion was 0.1 for both species. In the case of 80 % complex (Figure 4 C) the amplitudes of the correlation curves for both species are now clearly different and closely reflect the values expected from the simulations.

To check the existence of a ligand–receptor dynamic binding equilibrium, we used SCCF. The SCCFs for three bound fractions (20 %, 50 % and 80 % complex) are presented in Figure 4 D. The existence of an exchange dynamics is nicely highlighted by the presence of an amplitude of SCCF (colored lines), whereas the amplitude is zero for a static mixture (gray line). The SCCFs were fitted using the following model function assuming equal diffusion times for all species (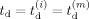) [Eq. (19)]:


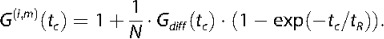
(19)

In Equation (19), *N* is the total number of labeled particles in the excitation volume and *t*_R_ is the relaxation time of the binding–unbinding process (the other parameters are defined as before). The overlay of SCCF and fit curves immediately shows that SCCF alone cannot distinguish between 20/80 % and 80/20 % mixtures, since SCCFs have equal amplitudes and relaxation times. But, of course, these two cases have different steady state concentrations of species which we can determine in the next step of our analysis (see Section 2.2.2).

#### 2.2.2. Global Analysis of SACF and SCCF

The SACF contains interrelated information on species concentrations and relaxation time whereas the SCCF contains additional selective information on the relaxation time. The kinetic term of SACF is scaled by the equilibrium constant, where it is not in the SCCF; that is, if the equilibrium is shifted to one side, the amplitude of the kinetic term of the two SACFs will vary characteristically, whereas it won′t change in the SCCF. Therefore global analysis of SACFs and SCCF will stabilize the fits significantly. If we express our model functions in terms of rate constants directly, global target fit of SCCF and SACFs will increase the accuracy of analysis and immediately will provide the values of rate constants. This is of particular interest for studying binding processes. To accomplish global target analysis we have to combine Equations (18) with (19) and replace *t*_R_ by (*k*_1,2_*+k*_2,1_)^−1^. Finally, we get the following model functions [Eqs. (20)]:


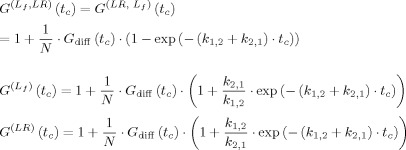
(20)

The results of global target fits for the example of the simulated dynamic binding equilibrium of a ligand as described in Section 2.2.1 (Figues 3 and 4) are compiled in Table 1. The relative deviation between simulated and fitted values ranges between 0.1 and 3 %. Given the fact that the obtained relative deviations are determined by the variations of simulated values and the confidence intervals of the fits, we computed by sampling of data subsets^50^ and extrapolation to larger photon numbers a theoretically expected error of less than 2 %. The excellent agreement with the simulated parameters in Table 1 confirms the high accuracy and precision of the applied analysis.

**Table 1:** Global target fit results of SACF and SCCFs for 50 % and 80 % formed complex in simulated dynamic binding equilibria (Figures 2 and 3)

Parameter		50 % bound				80 % bound		
	Simulated	Fit results		Rel. deviation [%]	Simulated	Fit results		Rel. deviation [%]
		Free ligand	Complex			Free ligand	Complex	
*N*^(*i*)^_FCS_	0.05; 0.05	0.0513	0.0507	2.6; 1.4	0.02; 0.08	0.0204	0.0797	2.0; 0.4
*t*_d_ [ms]	1.0	1.021		2.1	1.0	1.002		0.2
*k*_1,2_ [ms^−1^]	2.0	2.011		0.6	8.0	8.057		0.7
*k*_2,1_ [ms^−1^]	2.0	2.034		1.7	2.0	2.059		3.0
*N*_FCS_	0.1	0.102		2.0	0.1	0.1001		0.1

#### 2.2.3. Temporal Resolution of Polarization-Resolved fFCS to Detect the Conformational Exchange between Two States

**Figure 5:**
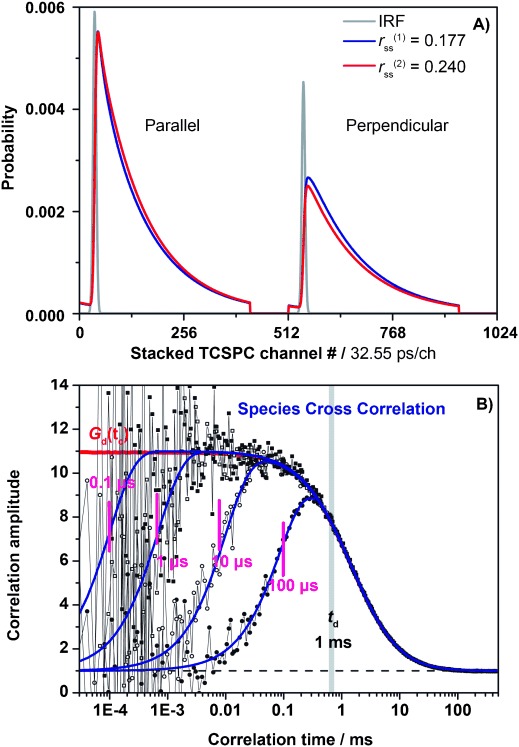
A) Conditional probabilities of polarization-resolved fluorescence decays used for fFCS. Total brightness *Q*_tot_=400 kHz, *τ*=4 ns, *N*=0.1, parameters of the observation volume are as in Section 2.2.1. The details on *r*(*t*) are given in the text. B) The species cross correlation functions [data (black circles: *t*_R_=100 μs, open black circles: *t*_R_=10 μs, black squares: *t*_R_=1 μs, open black squares: *t*_R_=0.1 μs), fits by Equation (19) (blue lines)] nicely recover the simulated parameters (magenta).

To explore the temporal resolution of polarization-resolved fFCS, we simulated typical polarization-resolved fluorescence decays of a single fluorescent dye attached to a macromolecule (Figure 5 A). The time-resolved anisotropy *r*(*t*) is described by three exponents [Eq. (21)]:


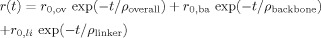
(21)

with the characteristic times for overall rotation (*ρ*_overall_), backbone fluctuations of the macromolecule (*ρ*_backbone_) and rotations around the linker *(ρ*_linker_), respectively, fractional initial anisotropy amplitudes *r*_0*,i*_. The steady-state anisotropy *r*_ss_ is computed by Equation (22):


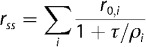
(22)

We simulated a case where the anisotropy senses the conformational dynamics of macromolecule as observed for a flexible loop in bacteriorhodopsin by Schröder et al.^51^ In the simulation, we studied a change of *ρ*_backbone_ due to the assumed conformational exchange in macromolecule, that is, state *1* characterized by a flexible backbone is in equilibrium with state *2* characterized by rigid backbone. In state 2 the effective fraction of the overall rotation is actually represented by the sum of fractional initial anisotropy amplitudes (*r*_0,ov_+*r*_0,ba_). In the simulation we assumed a fluorescence lifetime *τ*=4 ns and the following rotational correlation times *ρ_i_* (with corresponding fractional anisotropy amplitudes *r*_0*,i*_ ): *ρ*_overall_=20 ns (0.18); *ρ*_backbone_^(1)^=1 ns (0.10) and *ρ*_backbone_^(2)^=20 ns (0.10) for state 1 and *2*, respectively; *ρ*_linker_=0.3 ns (0.10). The conformational change results in a typical increase of the steady state anisotropy from *r*_ss_^(1)^=0.177 to *r*_ss_^(2)^=0.240. To find the temporal resolution limit we reduced the relaxation time for the exchange by factors of ten starting from 100 μs and ending at 100 ns. If the properties of the two states are known, the filter functions for conditional probabilities can be directly used to calculate the corresponding SCCFs shown in Figure 5 B. Analysing exchange between the two states by Equation (19), the simulated dynamics can be easily recovered over at least three orders of magnitude reaching a sub-microsecond time resolution.

Similar anisotropy differences in *r_ss_* are also observed if flexibly linked dyes stick temporally to the macromolecule, which may lead to complications in a quantitative analysis of FRET experiments.^52^ fFCS and anisotropy Photon Distribution Analysis (PDA)^53^ open up the possibility to detect heterogeneous dye environments and to characterize their exchange in an experiment, so that these results can be compared with detailed MD simulations.

### 2.3. Experimental Results for Single-Molecule FRET Experiments

One exciting experimental application of fFCS in biophysics is the study of protein conformational dynamics. As an example, we have analyzed Syntaxin 1 (Sx); Sx forms part of the so-called SNARE (soluble NSF attachment receptor) proteins that regulate synaptic vesicle release.^54^, ^55^ Sx is a transmembrane protein and the soluble domain consists of four long alpha helices that are known to undergo a conformational transition upon interacting with targets. One of the targets is SNAP25 located in presynaptic vesicles and it is known to open Syntaxin 1 allowing vesicle fusion and neurotransmitter release thereafter. On the other hand, Munc-18 is expected to act as a negative regulator of exocytosis.^55^, ^56^ However, Munc-18 is also essential for exocytosis and maybe it also works as an activator instead of inhibitor.^57^ Even when free in solution Sx had previously shown to be in equilibrium between two conformations,^34^ in the following referred to as “open” and “closed”. Sx has two domains, the Habc domain consists of a three-helix bundle and a flexible H3 domain has a single long helix. H3 associates with the Habc domain in the closed and dissociates in the open conformation.

**Figure 6:**
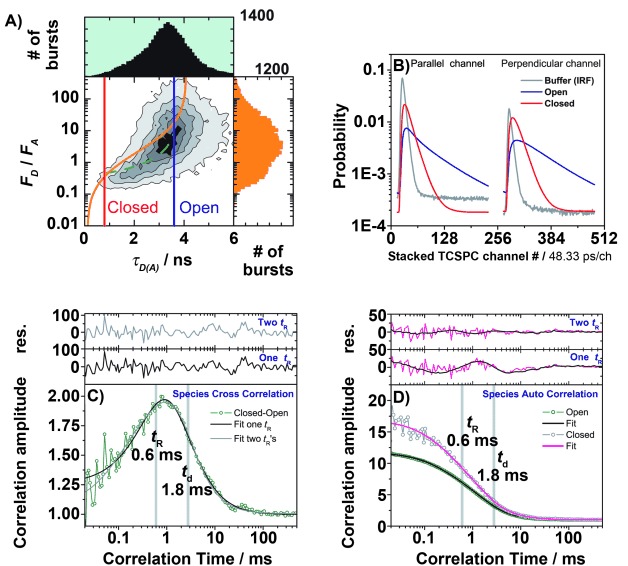
A) 2D Histogram *F*_D_/*F*_A_ versus *τ*_D(A)_ of Sx 105/225 labeled with Alexa488 and Alexa594. Grayscale contours represent binned accumulation of single-molecule events with the following parameters: The average green and red background count rates were 〈*B*_g_〉=4.4 kHz, 〈*B*_r_〉=0.39 kHz, respectively. An estimated 4.2 % of cross-talk signal was accounted for. The green over red detection efficiency (*g*_G_/*g*_R_) was 0.8 and the green quantum yield *Φ*_FD(0)_=0.8, red quantum yield *Φ*_FA_=0.4, D-Only lifetime *τ*_*D*(0)_=4.0 ns, and g-factors 0.995 and 1.378 for green and red channels respectively. On the histogram the main FRET population clearly shows a distribution that is off the static FRET line given by Equation (23) (orange). In dashed green, the dynamic FRET line Equation (24) shows the path taken by the conformational exchange. In this case there are two conformational states, plus a small fraction of D-Only. The open state was identified to have a lifetime *τ*_D(A)_^(open)^=3.6 ns and rotational correlation time of *ρ*=1.5 ns. For the closed state *τ*_D(A)_^(closed)^=0.8 ns. B) Conditional probabilities used for generating the filters: buffer (IRF), open [*τ*_D(A)_^(open)^=3.6 ns, *ρ*=1.5 ns] and closed [*τ*_D(A)_^(closed)^=0.8 ns, *ρ*=1.5 ns]. C) Species cross-correlation between closed and open states. Data is fit with Equation (19) and shows a relaxation time of *t*_R_=0.6 ms, and *t*_d_=1.8 ms. On top of this panel, residuals of fit with one or two relaxation times are shown. Additionally, we fit this SCCF globally with SACFs, shown in D), and the fit required two relaxation terms instead of one (*t*_R1_=1.1 ms, *t*_R2_=0.08 ms) with the same *t*_d_=1.8 ms. D) SACFs of the open and closed states as defined by the filters described above. Fits represent the global target fit of the two SACFs and the SCCF. The difference between global target fit of the SACF and SCCF with one and two relaxation times are shown on top of the SACF. The fit requires two relaxation times to reduce the characteristic deviations in the residuals. The SACFs fit with one relaxation time are not shown.

Herein, we used fFCS to extract the kinetic rates of this transition in a single-molecule FRET experiment combining lifetime and polarization-resolved information to maximize the contrast between conformations. The Sx double mutant (G105C/S225C) was labeled with donor dye Alexa488 and acceptor dye Alexa594 as described in Section 4.4.^34^ A solution of double-labeled protein was diluted to ∼10 pm in PBS and placed on the MFD setup (Section 4.3). After careful calibration of the detection efficiencies of our experimental setup, we performed an analysis of the single-molecule bursts at first. The results are displayed in two-dimensional frequency histograms of the FRET indicator *F*_D_*/F*_A_ (ratio of donor fluorescence over acceptor fluorescence)^49^ against fluorescence lifetime of the donor in the presence of acceptor, *τ*_D(A)_. In Figure 6 A, one can see a smeared population covering a broad range of fluorescence lifetimes. At least two FRET populations in addition to the third small D-Only contribution at high *F*_D_/*F*_A_ ratios with *τ*_D(0)_ of 4 ns can be detected (Figure 6 A). Such a presentation allows one to check for deviations from the static FRET line (orange line). The static FRET line in *F*_D_/*F*_A_ versus *τ*_D(A)_ plots is defined as [Eq. (23)]:


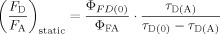
(23)

where *Φ*_*FD*(0)_ and *Φ_FA_* represent the quantum yields of the donor and acceptor fluorophores, respectively. The fluorescence lifetime of the donor without acceptor is *τ*_*D*(0)_ and *τ*_*D*(*A*)_ with an acceptor. As the populations are clearly off the static line, dynamics faster than the diffusion time is present.^58^ Then, given the simplest case of a dynamic two-state system, a FRET line for this exchange (green dashed line) can be traced that follows the form [Eq. (24)]:


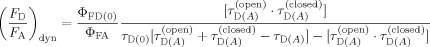
(24)

where *τ*_*D*(A),_ determined by the maximum likelihood estimator,^59^ corresponds to the fluorescence-weighted average lifetime (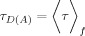). For further details on the analysis and interpretation of MFD histograms, we refer the reader to Sisamakis et al.^49^ The smeared population clearly follows the dynamic FRET line shown in green. The end points of the dynamic line correspond to *τ*_*D*(A)_^(open)^=3.6 ns and *τ*_*D*(A)_^(closed)^=0.8 ns, which were determined by sub-ensemble analysis of the dynamic FRET population. In combination with the average rotational correlation (*ρ*=1.5 ns, also reported in ref. 34) we computed two fluorescence decays with Equation (37) and the corresponding conditional probabilities (Figure 6 B), which were used to generate the filters according to Equation (11) using the global error minimization methodology described in Section 2.1. The SCCF computed by Equation (12) is presented in Figure 6 C. One can clearly see the enhanced negative correlation term as in the case of simulation data. When fitted with Equation (19) one recovers a relaxation time *t*_R_=0.6 ms which agrees nicely with values of *t*_R_=0.8 ms^34^ and *t*_R_=0.6 ms^49^ reported previously for another Sx double mutant (S91C/S225C), which also probes the exchange dynamics of the H3 domain with respect to the Habc domain.

In the previous section, we advised to use global target fit of the SACF and SCCF to stabilize the fit and reduce uncertainties. As indicated by the residuals of SACFs in Figure 6 D, the global target fit of Sx revealed the need of an additional relaxation time found in all three correlation functions simultaneously with following relaxation times and formal amplitudes: *t*_R1_=1.1 ms (84 %), *t*_R2_=0.08 ms (16 %). The *t*_R_=0.6 ms obtained with one free parameter recovers the average behavior, but only the global fit shows the need of the second faster relaxation time. The *t*_R2_=0.08 ms term may be caused by a more complex kinetic scheme for the conformational transition. For example, Reiner et al.^60^ have postulated that large-scale conformational motions require an intermediate structural unlocking step leading to a more flexible state which reacts to the final open state. Further measurements are needed to unambiguously solve this question. Finally, it is worth mentioning that the temporal resolution of fFCS is so powerful and provides such a good contrast that it is possible to distinguish additional kinetics not possible to observe otherwise, even with the 2D histograms from smFRET data.

If we assume an effective two state model for the open-closed transition, we can combine the results of the SACF and SCCF and extract the kinetic rates. Using the target fit Equation (20) with the average relaxation time of 0.6 ms, the individual rate constants *k*_1,2_=0.62 ms^−1^ and *k*_2,1_=0.94 ms^−1^ and the corresponding equilibrium constant *K*_*o*/*c*_=1.5 (*N*^(open)^=0.09, *N*^(closed)^=0.06) are obtained.

To conclude, Sx was selected as an experimental realization where FRET adds the additional spectral and lifetime information to maximize contrast in fFCS. The combination of the SACFs and SCCF has shown to be a powerful tool to extract the kinetic rates and the equilibrium constant between conformers.

## 3. Discussion

The introduction of lifetime filtering opened a door to increase contrast on FCS curves by selectively interrogating species rather than photons. In this paper we extend this idea to include polarization information to increase sensitivity. One could add also spectral information for selectivity as it will be discussed. During these studies several differences to previous implementations^22^–^24^, ^61^ were observed and are worth discussing.

### 3.1. Species Distinction by MFD: Generation of the Most Efficient Filters

One example is the simulation done in Section 2.1. In this case, the fluorescence of two species with the same fluorescence lifetime but small difference in their rotational correlation times (*ρ*^(1)^=0.1 ns and *ρ*^(2)^=0.3 ns) is studied. It was now the task of finding the most efficient filter. Scenario 1: single detector, single filter (^*1d–s*^*f*): If the signal is detected by a single detector (see Figures 1 A and 9 A, B) and the excitation is polarized, the polarization induced difference in the fluorescence decays is very small, so that conventional fluorescence lifetime correlation spectroscopy hardly accomplishes to separate the species (in Figure 1 A). Scenario 2: two detectors, independent filters (^*2d–in*^*f*): The separation is improved by using polarized excitation and polarization-resolved detection; one detector for the parallel and one for the perpendicular component (see Figures 1 B and 9 C, D). If the error minimization for the filters is done for each channel independently the separation of species improves, but the information on the relative intensities of polarized signals is lost. Scenario 3: two detectors, global filter (^*2d–gl*^f): Using polarized excitation and polarization-resolved detection, most efficient filters are generated by global error minimization (see Figures 1 C and 9 E, F) by properly maintaining the relationships between the parallel and perpendicular detection channels.

One way of judging the efficiency of the filters is to compare the quality of the computed SACFs by quantifying the noise by average normalized χ^2^ values with respect to the model functions. If we set the χ^2^ values of the most efficient method with global filter (^*2d–gl*^*f*) equal to one, the noise increases by a factor of 6.3 for independent filters (^*2d–in*^*f*) and even by a factor of 200 for a single filter (^*1d–s*^*f*). Moreover, we checked the capability of the filters to separate species by calculating the particle number *N* and the species brightness *Q=F*/*N*. The best agreement between simulated and obtained values was found for the global filter (^*2d–gl*^*f*). To conclude, all three approaches for fFCS work in principle but filters, which use the resolved signal from multiple detectors and are generated in a global way, are by far the most efficient.

**Figure 7:**
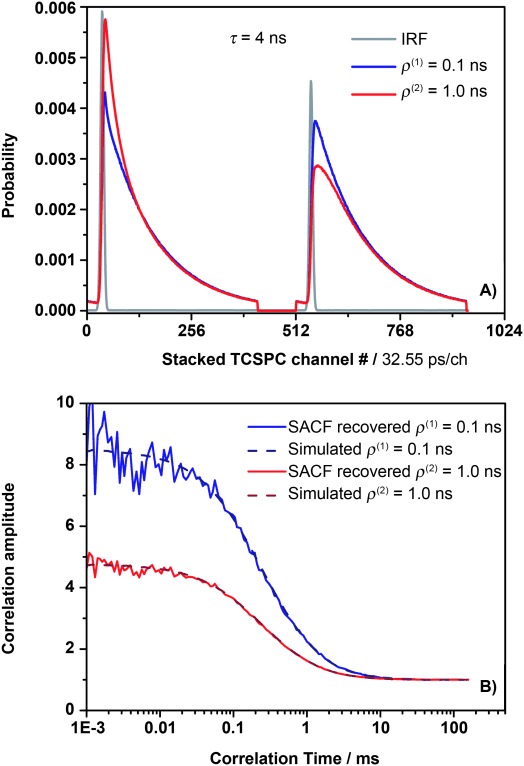
fFCS separates species when difference in rotational correlation time between two species is 0.9 ns whereas the fluorescence lifetimes are the same (4 ns). All other simulation conditions are the same as in Figure 1. The total duration of the simulated experiment is 1598 s. A) Conditional probability of the two species [species 1 *ρ*^(1)^=0.1 ns in blue and species 2 *ρ*^(2)^=1.0 ns in red]. IRF shape is shown in gray for reference. B) fFCS can separate species. SACF of species 1 and 2 are shown in blue and red respectively. The simulated species are shown as dashed lines for the same case, and represent the expected correlation functions. The noise is significantly reduced from the case presented in Figure 1.

In the following we discuss what features of the underlying species-specific fluorescence decays make the resulting filters most efficient for fFCS. The relative difference of the fluorescence parameters (e.g. *τ* or *ρ*) forms the basis to distinguish two species. Considering the currently used organic fluorophores and detectors in confocal microscopes, the fluorescence lifetime can range only between 0.2 and 6 ns, whereas the dynamic range of the rotational correlation time is significantly larger (0.2 and at least 30 ns), which offers a large dynamic range for the contrast. Furthermore, if we consider differences solely in the rotational correlation time, this difference should ideally affect the whole decay curve and not only a short segment as, for example, shown in Figure 7 A. In other words, if *ρ*^(1)^ and *ρ*^(2)^ ≪*τ*, the two decays are nearly equal for any time *t*≫*ρ* (Figure 9 E), no matter how different the two rotational correlation times are. This results in equal conditional probabilities to detect a photon characteristic for a certain species and thus limits the discrimination efficiency. We demonstrate this effect by comparing two simulations, where the rotational correlation time of species 1 is kept constant (*ρ*^(1)^=0.1 ns) and the rotational correlation time of species 2 is either *ρ*^(2)^=0.3 ns (Figure 9 E) or *ρ*^(2)^=1.0 ns (Figure 7 A). The low noise of SACF in Figure 7 B with *ρ*^(2)^=1.0 ns is consistent with drop of the average normalized χ^*2*^-value by a factor of 100 as compared to SACF with *ρ*^(2)^=0.3 ns (Figure 1 C).

In cases where the differences of a chosen parameter (e.g. rotational correlation or lifetime) are not large enough, the addition of another experimental observable can be used to increase contrast. For example, one can combine changes in lifetimes, rotational correlation times and spectral information (like FRET) to be more precise in the selection.^33^ In this case up to four decay curves (two for donor and acceptor, respectively) can be combined in a global filter to maximize contrast and thereby minimize noise.

### 3.2. Influence of the Filter Quality

**Figure 8:**
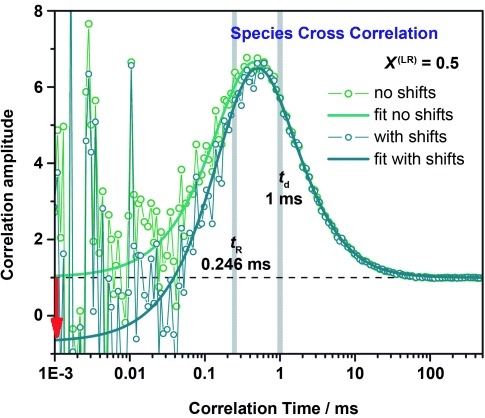
Overlay of SCCFs calculated by fFCS for dynamic mixture of species with *X*^(LR)^=0.5 [free ligand with *ρ*^(free ligand)^=2.71 ns and complex with *ρ*^(complex)^=5.18 ns] in buffer without (light green) and with shift (dark green) of species decay curves relative to IRF. Further simulation conditions are given in Figure 3. Shifts of patterns are 0.3 ns and 0.2 ns for the detection channels of parallel and perpendicular polarization, respectively.

To make a precise selection, there are some experimental requirements and limitations that are worth discussing. It is well known^45^ that the response function of avalanche photodiodes (APDs) exhibits a noticeable dependence on the photon count rate, which can distort the decay patterns for individual species. This could lead to small time shifts on the measured fluorescence decay of the mixture compared to the decay of individual species. We observe this shift in measurements with a mean particle number *N*=0.1, because under these conditions the count rate is much higher than under the conditions where we measure the IRF by using the Raman and Raleigh scattered photons of the pure buffer solution. In Figure 8 we show two SCCF and their corresponding fit curves to demonstrate how the shift by 0.3 ns and 0.2 ns in parallel and perpendicular detection channel, respectively, can affect the baseline in the correlation curve. At shorter correlation times the SCCF curve with shifted decay patterns enters the range of negative correlation amplitudes which makes no scientific sense. However, the relaxation times in these two cases are not affected. This outcome is quite positive, because it means that if we allow the fit to enter into negative correlation amplitudes range we can still recover the relaxation rate between the two species.

Another problem is to find appropriate filters that describe each separated species. In many cases the properties of the individual states can be determined by shifting the equilibrium towards one side, for example by adding a binding partner or stabilizing/destabilizing the macromolecule. In the case of slow dynamics it might be possible to resolve the species in 2D plots and generate sub-ensemble decay histograms.^61^ Moreover, a set of ensemble measurements with high photon statistics will also help to judge the heterogeneity of the sample. These results can be then compared with other methods such as 2D plots and PDA which are very useful to detect structural dynamics by FRET.^58^ If slightly wrong decay curves are used to generate filters for fFCS, the amplitudes of SACF and SCCF become distorted but so far we can still recover correct relaxation times.

### 3.3 Strategy to Eliminate Detector Afterpulsing Effects in Mixtures

Another experimental artifact is the occurrence of highly correlated events referred to as afterpulsing of the detectors up to the time range of 10 μs. It has been shown by Enderlein^23^ that FLCS is able to correct the correlation curves of a single species for afterpulsing in a single detection channel by adding an uniform baseline as a model for “signal” generated by afterpulsing. In our experience, this technique does not work well for the case of multiple species mixture. Thus, SCCF and SACF from real data will be affected by afterpulsing in the short correlation times range (less than 10 μs). According to ref. 62 we can avoid the afterpulsing correlation and detector dead time effects by splitting the fluorescence signal between two independent detectors and cross-correlating these two signals. This requires the addition of a 50/50 beam splitter in our parallel and perpendicular detection channels. The theory described in Section 2.1 is the same, but we need to correlate the signals of one parallel/perpendicular detector pair with the corresponding signal from second parallel/perpendicular detector pair using their corresponding filter functions for each pair of detectors. In total four green detectors, instead of two green detectors in a typical MFD setup, have to be used. With this solution one could increase the temporal resolution to below μs. This implementation and the exploitation of this powerful technique are left for further studies.

As a final remark we want to give a recommendation for optimal measurement conditions of fFCS. Due to the filter action, the effective (“useful”) signal is significantly lower than the raw signal so that the noise levels of all filtered FCS curves for given experimental conditions are significantly higher than that of conventional FCS curves. If the average number of molecules in observation volume is chosen too high, the noise level of the SACFs and SCCF becomes comparable to the amplitudes of diffusion and kinetic terms of the correlation function and the analysis becomes ambiguous. This problem can be avoided by using concentrations, which are optimal for fFCS measurements with an average number of molecules *N*≤0.1, which was also chosen in simulations. This concentration is slightly lower than for standard FCS, because the background signal is anyhow filtered out.

## 4. Theory and Methods

### 4.1 Theory for Independent Error Minimization (Scenario 2 of Filter Generation)

TCSPC decay histograms {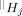} and {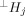} of a mixture of two species (*i*=1; 2) can be written as a superposition of species histograms in the form of [Eq. (25)]:


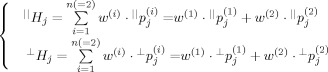
(25)

where 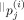 and 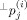 are the normalized probabilities of counting a photon within the *j*th TCSPC channel for species *i* in parallel (||) and perpendicular (⊥) polarizations relative to linearly polarized excitation laser beam respectively. To compute the normalized probabilities we follow ref. 22 for a single detection channel where the counts in the TCSPC histograms {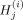} were normalized by the total number of photons [Eq. (26)]:


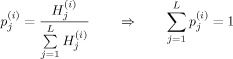
(26)

with *i*=1, 2; *j*=1;..; *L*; and *L* is the number of histogram bins or TCSPC channels. In the case of two detectors, one parallel and one perpendicular, the independently normalized probabilities, 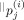 and 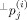, are [Eq. (27)]:


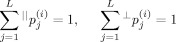
(27)

In Equation (25) 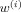 is the number of photons corresponding to the *i*th species. To increase selectivity, Equation (25) could be expanded to account for spectral differences (color detection) by including a summation over the number of color channels.

Two filter sets 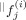 and 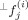, with *i*=1; 2, can be derived to satisfy the relationships [Eqs. (28)]:


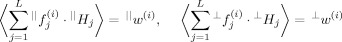
(28)

where 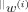 and 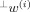 are the photon counts of the *i*th species in parallel and perpendicular detection channels, respectively (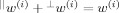). Brackets represent averaging over an infinite ensemble of measurements. The relative errors in both detection channels are simultaneously independently minimized like [Eq. (29)]:


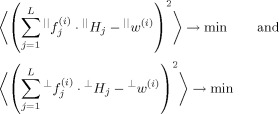
(29)

then 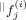 and 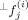 are expressed with the help of the weighted pseudoinverse of the matrices 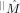 and 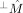, (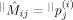 and 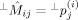), as^43^ [Eq. (30)]:


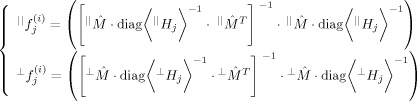
(30)

since single-photon detection in a TCSPC channel follows Poissonian statistics. In Equation (30), 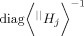 and 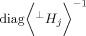 are 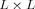-dimensional diagonal matrices with diagonal elements 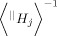 and 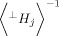, *j*=1;…; *L*, and *T* represents the transpose operator. It can be shown that 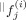, 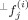 and the patterns 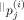, 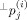 are orthonormal systems [Eq. (31)]:


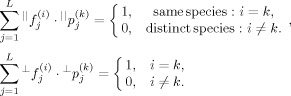
(31)

With the filters 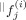 and 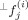 it is now possible to separate the correlation curves of two molecular species independently. The fFCS functions between perpendicular and parallel fluorescence signals 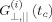 are defined by Equation (32):


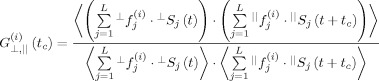
(32)

where 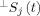 is the perpendicular signal in the *j*th TCSPC channel at measurement time *t* and 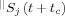 is the parallel signal in the *j*th TCSPC channel at measurement time 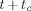. The polarization cross-correlation functions of a single species *i* 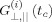 and 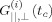, averaged over an infinite number of measurements or over sufficiently long time (≫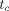), can be written as [Eq. (33)]:


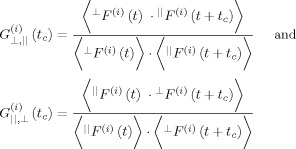
(33)

where 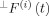 and 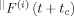 are pure fluorescence signals from the *i*th-type molecular species (summed over all TCSPC channels). The global fit of two correlation curves significantly reduces the statistical errors and makes fit results more accurate.

To show how to calculate the filters according to the theory presented we use the simulated mixture introduced already in Section 2.1. A mixture of two fluorescent species with the same (4 ns) lifetime, but different characteristic rotational correlation times (0.1 ns and 0.3 ns) in water are simulated.

**Figure 9:**
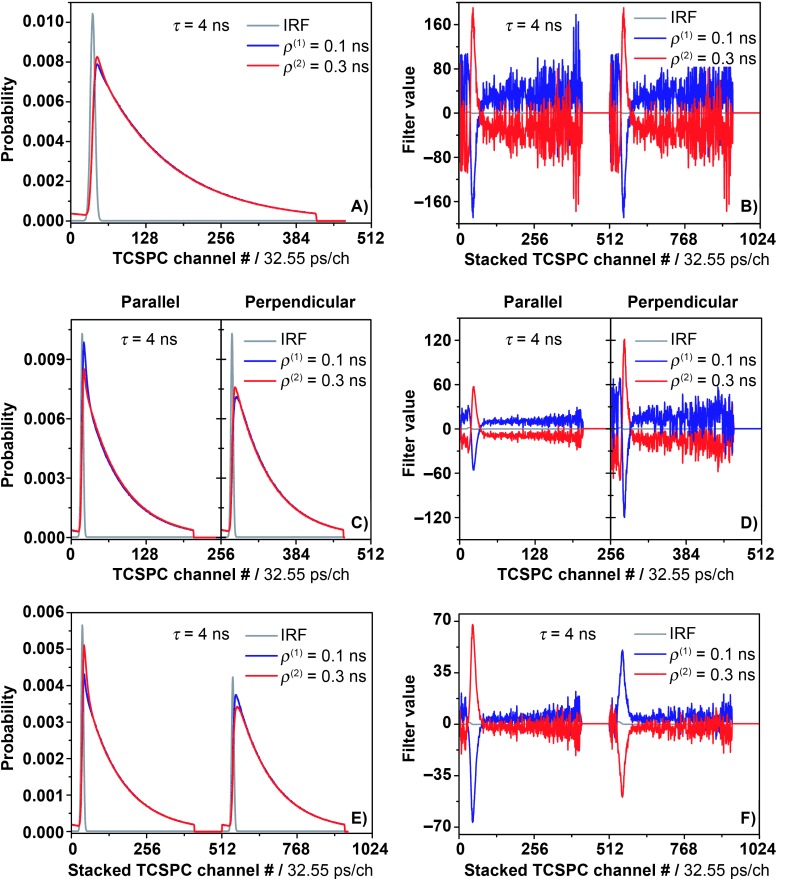
Decays and filters for simulated mixture of two species with same fluorescence lifetime but different rotational correlation times. The parameters used for each species in the simulation are *Q*^(1)^=*Q*^(2)^=150 kHz, *t*_d_^(1)^=*t*_d_^(2)^=0.25 ms, *N*^(1)^=0.133, *N*^(2)^=0.266, dark counts=0.2 kHz, scatter=2.5 kHz. The lifetime of both species is the same (*τ*_G_^(1)^=*τ*_G_^(2)^=4.0 ns), and the respective rotational correlation times are *ρ*^(1)^=0.1 ns, *ρ*^(2)^=0.3 ns. The total duration of the simulated experiment is 2479 s. A) The normalized TCSPC histograms (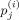) for each species as described in Equation (26). The IRF shape is shown in gray. The first species (blue line) has *ρ*^(1)^=0.1 ns and the second species (red line) has *ρ*^(2)^=0.3 ns. B) The generated filters for each species (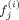) are plotted according to the notation: IRF shown in gray, species 1 in blue, and species 2 in red. C) The independently normalized TCSPC histograms for species 1 and 2 according to Equation (27). D) Simultaneously independently minimized filters using Equations (29) and (30). E) Conditional probabilities for each species as described in Equations (4)–(6). The IRF shape is shown in gray. The first species has a rotational correlation of *ρ*^(1)^=0.1 ns and its decay is shown in blue. The second species with rotational correlation *ρ*^(2)^=0.3 ns is shown in red. F) The filters generated for each species (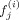) according to Equations (10) and (11) are plotted. The IRF filter is shown in gray, species 1 in blue, and species 2 in red.

Given polarized excitation and single detection channel at magic angle conditions (54.7°) separation of species with the same lifetime is impossible. Even in MFD, using the total decay histograms as 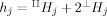 (equivalent to magic angle conditions) does not provide separation of species. However, if unpolarized detection (assuming 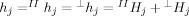) is used, one would expect small differences between TCSPC decay histograms of two species. Figure 9 A, B shows the stepwise generation of the filters for this case imitating single detection channel experiment.

First, the conditional probabilities are required for each species [Eq. (26)]. These can be generated independently by measuring fluorescence decays (Figures 9 A,C,F) by TCSPC with pure concentrated solutions of each species (≥25 nm; on average about 30 molecules in a 2 fl detection volume). However, if the species are not available separately, it is also possible to compute the theoretical decay patterns based on the expected fluorescence lifetimes and rotational correlation times. This can be accomplished by measuring the instrument response function (IRF), convoluting it with the expected fluorescence decay for each species and normalizing these histograms using Equations (4)–(6), (26), (27). The single detection channel filters are then obtained by minimizing the errors according to Equations (11), (29). The blue line corresponds to species 1 with *ρ*^(1)^=0.1 ns and the red line corresponds to species 2 with *ρ*^(2)^=0.3 ns. Finally, the SACFs [Eq. (12)] for the case of a single detection channel, with no split in polarization, using the filters shown in Figure 9 B, are calculated (Figure 1 A). There is poor separation of species and their correlations (blue and red lines for the species *ρ*^(1)^=0.1 ns and *ρ*^(2)^=0.3 ns respectively) are very noisy.

The separation of species gets better when the two polarized decay components are considered as shown in Figures 9 C,E. In Figure 9 C, we can see already the advantage of measuring the distinct orientations. The normalized TCSPC decays are distinct due to 0.2 ns difference in the rotational correlation times between species. For two independent detection channels the error minimization described by Equation (29) is performed. These filters are then used to calculate the SACFs for species by Equation (12). The independent filters also were stacked and applied to the raw data in a modified format where the TCSPC channels of both detectors are stacked in a single array with length 2*L*. This correlation curves show better separation of species than in the single detector case.

Figure 9 E shows conditional probabilities for each species as described in Equations (4)–(6). The IRF shape is shown in gray. The first species has a rotational correlation of *ρ*^(1)^=0.1 ns and its decay is shown in blue. The second species with rotational correlation *ρ*^(2)^=0.3 ns is shown in red. The filters generated for each species (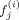) according to Equations (10), (11) are plotted in Figure 9 F. The IRF filter is shown in gray, species 1 in blue, and species 2 in red. Calculated by Equation (12) SACFs are shown in solid blue and red lines for species 1 and 2, respectively. For comparison, the simulated correlation functions are shown as dashed lines in dark blue and wine for species 1 and 2. The separation of species is obvious to the eye, and the estimated error in the recovered parameters is within 2 %. In all cases, we make sure that exactly the same number of photons are used and the quality of separation is defined only by the different filters.

### 4.2 Data Generator

Our simulations were done by the Brownian dynamics approach^63^–^66^ with an own in-house C++ program. The spatial intensity distribution of the observation volume was assumed to be a 3D Gaussian.^67^ In previous implementations, Brownian diffusion was modeled in a box with periodic boundary conditions.^63^ We have developed our own approach, where “molecules” diffuse freely in an “open” volume. In this way, we gain several advantages: i) the efficiency of the algorithm is improved for a given box size; ii) a fractional number of molecules can be realized, which is important for irreversible reactions; and iii) the simulation speed can be improved because the calculations can be easily parallelized.

In our simulation of an “open” volume, molecules are allowed to leave the simulation volume, after which they are not further tracked. To keep the desired average number of molecules constant, new molecules are added at each time step as described below.

Analog to the 3D Gaussian observation volume in Equation (3), the simulation volume is defined by a surface 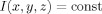, which for our observation volume can be approximated by an ellipsoid [Eq. (34)]:


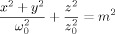
(34)

with *m*=10 unless stated otherwise. One can show that the average number of molecules entering the simulation volume per time step Δ*t* is given by Equation (35):


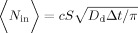
(35)

while the distribution of distances Δ*l* of new molecules from the ellipsoid surface Equation (34) is [Eq. (36)]:


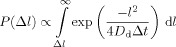
(36)

In Equations (35) and (36), *c* stands for the concentration of the molecules, *S* is the surface area of the ellipsoid Equation (34), and *D*_d_ stands for the diffusion coefficient. Equations (35), (36) can be obtained by solving the diffusion equation with initial conditions *c*(*l*<0, *t*=0)=*c* and *c*(*l*≥0, *t*=0)=0. The curvature of the surface Equation (34) is thereby neglected, which requires that the time step Δ*t* is small (i.e. *D*_d_Δ*t*≪*m*ω_0_). The number of new molecules per time step (Δ*t*=0.005 ms) was generated according to a Poisson distribution with a mean value given by Equation (35). Test simulations have shown that the number of molecules calculated by FCS analysis Equation (1) of simulated data agree with the assumed values within 0.2–0.5 %. Note also that the average number of molecules in the simulation volume can be fractional, which is not the case for a box with periodic boundary conditions. As a result, all concentration values are possible irrespective of the simulation volume size.

Our approach allows also for straightforward and unbiased modeling of photobleaching and other irreversible reactions: when bleached molecules leave the simulation volume, new “bright” molecules are automatically added, Equations (35) and (36). Finally, the trajectories of molecules in “open volume” are not endless, which allows efficient distribution of calculations between several CPUs.

To model diffusion, normally distributed random numbers Δ*x*, Δ*y* and Δ*z* were added to the *x*, *y* and *z* coordinates of each molecule, respectively, at each time step Δ*t*, where 〈Δ*x*^2^〉=〈Δ*y*^2^〉=〈Δ*z*^2^〉=2*D_d_*Δ*t*. The average number of photons emitted by a molecule and registered by the *i*th detector at each time step is 
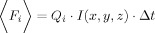
, where *Q_i_* stands for the brightness of the molecule as measured in the *i*th detection channel.

Binding or conformational dynamics were modeled by allowing states 1→2 and 2→1 transitions. The initial fractions of molecules in states 1 and 2 were *k*_2,1_/(*k*_1,2_+*k*_2,1_) and *k*_1,2_/(*k*_1,2_+*k*_2,1_), respectively. The times the molecules spent in states 1 and 2 (*t*_1_ and *t*_2_, respectively) were exponentially distributed with *P*(*t*_1_)=*k*_1,2_^−1^exp(−*k*_1,2_*t*_1_) and *P*(*t*_2_)=*k*_2,1_^−1^exp(−*k*_2,1_*t*_2_). The diffusion times of the species are given for each case and the time step was 0.005 ms for all simulations. Background was added when necessary.

TCSPC data were generated as proposed by Chowdhury et al.,^68^ assuming a Gaussian excitation pulse profile with an FWHM of 0.3 ns. The parallel and perpendicular decay components *F*_||_(*t*) and *F*_⊥_(*t*) were modeled as [Eq. (37)]:


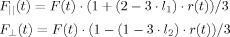
(37)

In Equation (37), *F*(*t*) is the fluorescence intensity decay (typically mono-exponential with *τ*=4 ns), *l*_1_=0.0308 and *l*_2_=0.0368 are correction factors,^48^, ^69^ and the anisotropy decay *r*(*t*) is either single-exponential or given by Equation (21). The background signal consists of dark counts (uniformly distributed over the TCSPC channels) and a scatter contribution [repeating instrument response function (IRF) of the setup]. The simulated data were saved in the data format of SPC-132 TCSPC cards (Becker & Hickl GmbH, Berlin, Germany) and were suitable for direct software testing. The algorithm was thoroughly tested by analyzing the simulated data by the FCS, FIDA, and BIFL^9^, ^16^, ^45^, ^64^ methods.

### 4.3. MFD Experiments

The MFD setup used for single-molecule FRET detection was previously described.^32^ In short, the active mode-locked argon-ion laser was operated at 477 nm (28 kW cm^−2^). A dual-band dichroic mirror 488/594PC (AHF Analysentechnik, Tübingen, Germany) was used to separate excitation light from fluorescence. The detection spectral windows for the donor (D) and the acceptor (A) were defined by bandpass filters HQ535/50 and HQ650/75 (AHF Analysentechnik) respectively. Burst integrated lifetime analysis using the signal of two green and two red detectors split by polarization were detected by avalanche photodiodes (AQ 151, EG&G (Vaudreuil, Quebec, Canada)) coupled to a counting board (SPC432, Becker & Hickl, Berlin) and a personal computer. Single-molecules bursts were selected, analyzed and used to generate the corresponding sub-ensemble TCSPC histograms for the mixture and the MFD 2D histograms.

### 4.4. Molecules

Syntaxin-1 was expressed from pET28a in the BL21 (DE3) strain of Escherichia coli as previously reported.^70^, ^71^ The recombinant Sx contained N-terminal His6 tags that served to affinity purify it on nickel-nitrilotriacetic acid-agarose (Qiagen). After affinity purification, DTT (5 mm) and EDTA (2 mm) were added. The tags were cleaved off by thrombin. Cleavage occurred overnight during concomitant dialysis (NaCl (50 mm), Tris HCl (20 mm, pH 7.4) and DTT (1 mm)). Subsequently, Sx was purified with MonoQ column using the Äkta Explorer (Amersham Pharmacia Biotech). Collected fractions were analyzed by SDS/PAGE and Coomassie staining with 95 % purity. Before labeling, DTT was removed from the proteins by gel filtration using PD-10 columns and PBS as elution buffer (Na_2_HPO_4_ (10.4 mm), KH_2_PO_4_ (3.2 mm) and NaCl (123 mm)).

Random labeling of Sx was accomplished by adding a mixture of Alexa 488- and Alexa 594-maleimide dyes (Molecular Probes) with a ratio of 10:30 vs. protein concentration respectively in order to obtain an 1:1 labeling. After 3–5 hr of reaction on ice, labeling was stopped by adding DTT (10 mm). The unreacted dye was removed via gel filtration using a PD-10, followed by extensive dialysis against PBS (Na_2_HPO_4_ (10.4 mm), KH_2_PO_4_ (3.2 mm) and NaCl (123 mm)) containing DTT (1 mm) and SM-2 Biobeads (Bio-Rad). All measurements were done in PBS buffer previously cleaned by charcoal.

## 5. Conclusions

fFCS extends the previously presented FLCS^22^–^24^, ^61^ by including the anisotropy and spectral information for improved species separation. With some modification, fFCS is capable of discriminating detector afterpulsing from fluorescence signal as has been described in Section 3 as well as removing effect of the scatter photons on the correlation amplitude (Section 2). More importantly, we have shown that fFCS is able to distinguish species even when they have very close or even the same fluorescence lifetime using just differences in time-resolved fluorescence anisotropy, with an estimated error of <2 %. In addition, to increase selectivity, one can combine parameters such as lifetime, spectral window and anisotropy. One example is the protein Synatxin 1, which shows conformational dynamics in solution between open and closed conformation. A relaxation time of 0.6 ms recovered by fFCS agrees with previous results.

One has to bear in mind that there are different error minimizations forms and this is totally different from the previously presented lifetime FCS.^22^ Given the particular case, it is important to consider the differences on minimizations procedures.

Finally, standard tools such as FCS and FCCS could not even distinguish the static binding from dynamic one if there is no brightness change upon binding, as shown in Section 2, and fFCS now opens a window to study kinetic mechanisms in detail and we foresee many possible applications: *1) Removal of background*: In experiments at very low molecular concentrations and for measurements in highly scattering media, fFCS can be used as tool to eliminate the influence of a scattered background on the FCS curves. Usually, the drop of amplitude is corrected by using a formula of Koppel.^14^ However, we have shown this correction works only to a limited extent,^16^ whereas fFCS fully recovers the amplitudes, which is crucial for a proper concentration determination. Also in FRET-FCS experiments with a dynamic equilibrium the correct extraction of amplitudes or species concentrations is the prerequisite to determine the equilibrium constant. *2) Selective correlation in mixture*: fFCS can be applied as a tool to compute species-specific correlation curves from mixture of different species, provided that a separation could be done based on lifetime or/and anisotropy or/and spectrum. *3) Analysis of interconversion kinetics between species*: In our group, SCCF and SACF have developed as a hallmark for kinetics, which also allows for the assignment of the originating states. The applications can be split into two general groups: i) Experiments with single-color detection based on anisotropy changes due to ligand binding (static or dynamic), homo-FRET and mobility changes of a dye coupled to a biomolecule (e.g. dye sticking observed in FRET studies). ii) Experiments with multicolor excitation and detection based on lifetime and anisotropy changes of species (in all single-molecule FRET studies of biomolecules with MFD technique). In this case, differences in the FRET efficiencies of the species resulting in different green and red signal levels make it possible to include the spectral heterogeneity as a third parameter for species selection.
